# The A3 Problem Solving Report: A 10-Step Scientific Method to Execute Performance Improvements in an Academic Research Vivarium

**DOI:** 10.1371/journal.pone.0076833

**Published:** 2013-10-29

**Authors:** James A. Bassuk, Ida M. Washington

**Affiliations:** 1 Department of Research Continuous Performance Improvement, Seattle Children's Research Institute, Seattle, Washington, United States of America; 2 Office of Animal Care, Seattle Children's Research Institute, Seattle, Washington, United States of America; University of Central Florida, United States of America

## Abstract

The purpose of this study was to illustrate the application of A3 Problem Solving Reports of the Toyota Production System to our research vivarium through the methodology of Continuous Performance Improvement, a lean approach to healthcare management at Seattle Children's (Hospital, Research Institute, Foundation). The Report format is described within the perspective of a 10-step scientific method designed to realize measurable improvements of Issues identified by the Report's Author, Sponsor and Coach. The 10-step method (Issue, Background, Current Condition, Goal, Root Cause, Target Condition, Countermeasures, Implementation Plan, Test, and Follow-up) was shown to align with Shewhart's Plan-Do-Check-Act process improvement cycle in a manner that allowed for quantitative analysis of the Countermeasure's outcomes and of Testing results. During fiscal year 2012, 9 A3 Problem Solving Reports were completed in the vivarium under the teaching and coaching system implemented by the Research Institute. Two of the 9 reports are described herein. Report #1 addressed the issue of the vivarium's veterinarian not being able to provide input into sick animal cases during the work day, while report #7 tackled the lack of a standard in keeping track of weekend/holiday animal health inspections. In each Report, a measurable Goal that established the basis for improvement recognition was present. A Five Whys analysis identified the Root Cause for Report #1 as historical work patterns that existed before the veterinarian was hired on and that modern electronic communication tools had not been implemented. The same analysis identified the Root Cause for Report #7 as the vivarium had never standardized the process for weekend/holiday checks. Successful outcomes for both Reports were obtained and validated by robust audit plans. The collective data indicate that vivarium staff acquired a disciplined way of reporting on, as well as solving, problems in a manner consistent with high level A3 Thinking.

## Introduction

Problem solving tools come in many shapes and sizes. From a complex, multipage research grant application designed to unravel the molecular mechanisms of human disease [1], [2], to the one-page A3 Report developed by the Toyota Motor Corporation [3], [4], problem solving tools typically have the scientific hypothesis as the one common attribute. A3 Reports exist as the following 3 types: *(i)* The Problem Solving A3 Report, *(ii)* The Proposal A3 Report, and *(iii)* The Status A3 Report [4].

Originally developed on A3 paper (297×420 mm, 11.69×16.54 in), the largest size that can fit through a fax machine, the A3 Problem Solving Report fully documents a given process on one side of one sheet of paper. Based on the 13^th^ Principle of the Toyota Way (“Make Decisions Slowly by Consensus”) [3], the A3 Problem Solving Report is a tool that describes how consensus on complex decisions can be efficiently reached. The key to generating a good A3 Report is *nemawashi* – the process of getting consensus. The purpose of A3 Reports has been described as written documents to support mentor/mentee dialogues during application of the improvement kata [5]. Excellent textbooks have been written that provide expert A3 advice and insights, especially in A3 Thinking [4], [6], [7].

A3 Reports are based on the Plan-Do-Check-Act cycle, a high level problem solving algorithm pioneered by Walter Shewhart in the 1930s [8] and later adopted by W. Edwards Deming in the 1950s [9]. The PDCA cycle has evolved into the Plan-Do-Study-Act (PDSA) cycle and has recently been reviewed [10].

Performance improvement (“lean”) initiatives in a non-profit research organization are being championed by Seattle Children's Research Institute (SCRI), a multicenter complex founded in 2006 by Seattle Children's Hospital. The early adapter of lean at the Institute was the Office of Animal Care (OAC), which oversees an accredited vivarium facility that supports dozens of laboratories through approved animal use protocols. Such accreditation has been granted by the Association for Assessment and Accreditation of Laboratory Animal Care International. Using the same tools and methods of the Toyota Production System [11], the OAC reported that the elimination of wasteful procedural steps in the dirty cage wash area led to marked improvements in material flow, macroenvironmental quality, increased employee safety and enhanced customer service [12]. Despite being reported by Seattle Children's research leaders [13], [14], these improvements were not sustained when the vivarium relocated from a temporary research facility to its permanent home in downtown Seattle.

Another academic research vivarium engaged in performance improvement via lean thinking is the Center for Comparative Medicine at Massachusetts General Hospital, who introduced their management of animal facility operations using the Toyota Production System approach to the 2005 Annual Meeting of the American Association for Laboratory Animal Science [15]. Waste removal and process improvements have converted the Center from operating in a deficit to annually realizing a small profit [16].

Two problems that the OAC chose to examine are of importance to all vivariums due to their impact on animal welfare. The first problem addressed involvement of the veterinarian during the OAC response to sick animals. The second problem focused on the potpourri of inefficient methods that OAC staff employed on weekends and holidays to keep track of animal health checks. The current publication describes how the OAC utilized A3 Thinking to drive A3 Problem Solving Reports to completion. Specific attention is drawn to the increase in the level of quality of its animal care and associated services while simultaneously removing waste from its system.

## Materials and Methods

### I. Human Subject Research

The study presented in this manuscript did not perform any research that used, created, or shared Protected Health Information. The study was therefore not subject to the State of Washington Uniform Health Information Act or the United States of America Health Insurance Portability and Accountability Act.

### II. The A3 form

**Figure 1:**
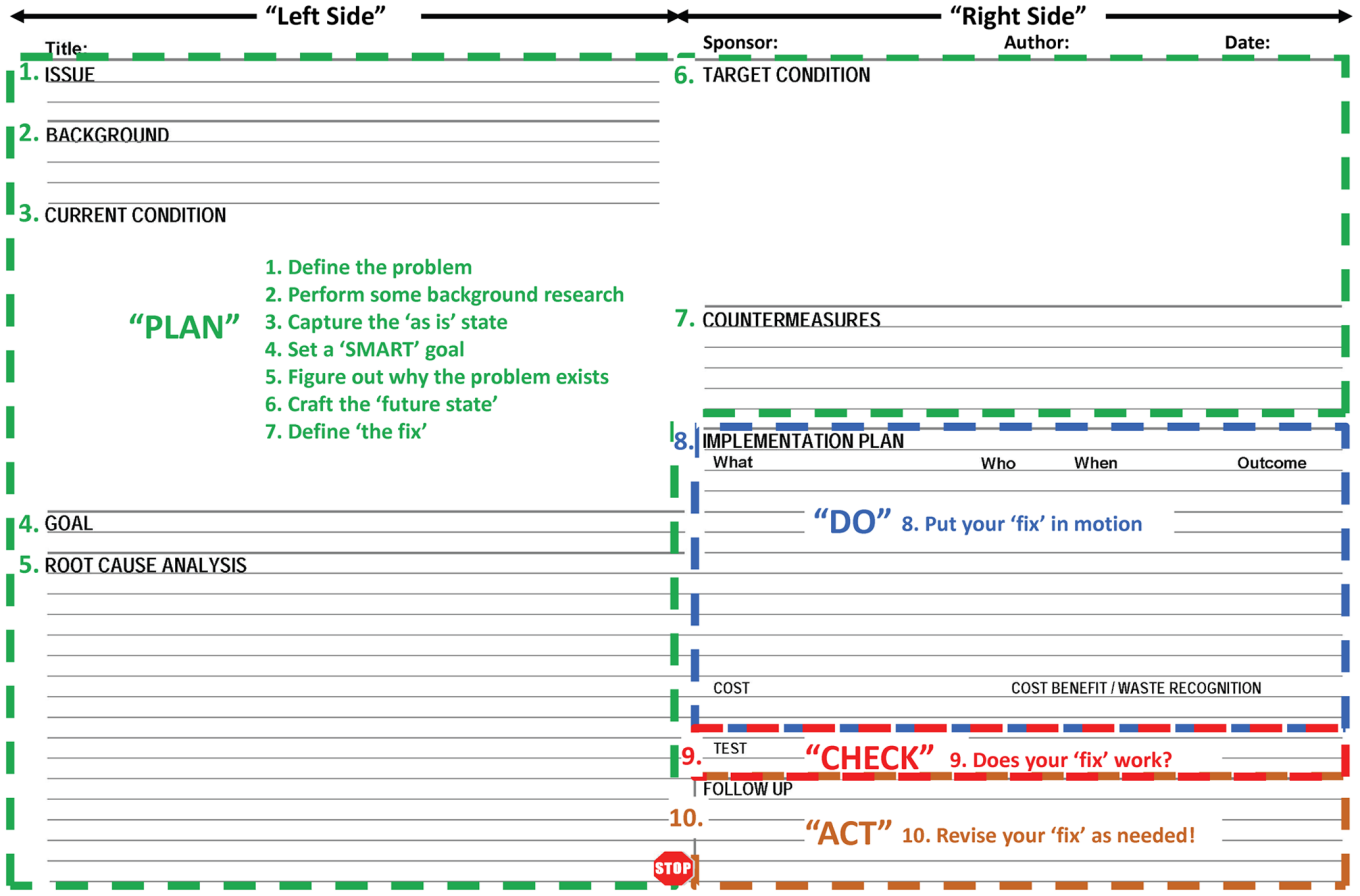
The A3 Problem Solving Report: A 10-step scientific method to help solve problems. The A3 form is printed on 11×17 inch paper, is filled out with a pencil, and contains 10 sections, as illustrated above. The Goal statement is a hypothesis which is “checked” via the Test (step 9) and auditing (step 10).

The blank A3 Problem Solving Tool form used at Seattle Children's Research Institute is populated on both sides of 11×17 inch paper. The front side of the form consists of a left and right side, and has 10 sections which are listed below and illustrated in Figure 1. Sponsor approval lines are provided for signoff once the left and right sides have each been sequentially completed.

Step 1. Issue. A clear, focused, stand-alone statement that defines the problem.

Step 2. Background. Details that cannot be described in the Current Condition drawing and useful baseline metrics may be included in this section.

Step 3. Current Condition. A drawing that conveys a complete understanding of the current situation is essential in order to realize what improvements may be necessary.

Step 4. Goal. A quantitative statement that will form the basis for how improvement will be measured is provided in this section. In many ways, this statement is the hypothesis for the experiment. Goals in this context are equivalent to Targets, which need to be SMART (**s**pecific, **m**easurable, **a**ttainable, **r**elevant and **t**imely) [17].

Step 5. Root Cause Analysis. The Root Cause Analysis section can accommodate either a Five Whys analysis or a Ishikawa (fishbone) diagram [18]. These two options give the Author flexibility, depending on the complexity of the problem.

Step 6. Target Condition. A drawing that conveys a complete understanding of what the situation will look like once the improvements have taken hold is placed on this step. The Target Condition describes what is necessary to meet the Goal.

Step 7. Countermeasures. A listing of the improvements needed to attain the Target Condition.

Step 8. Implementation and Cost Analysis. A listing of specific tasks that will lead to improvements, along with timelines, ownership and the expected outcomes is described in this step. The expected outcomes are an essential component of the scientific process because they provide the basis for evaluating whether or not the improvements are successful. This section also contains the cost of completing the A3 report, how much money will be saved after implementation of the Countermeasures, and what types of waste have been removed from the process.

Step 9. Test. A small pilot conducted over 1–2 weeks is recommended. Do the measured results match the predicted results?

Step 10. Follow Up/Audit. This section contains a description of an audit plan (typically 30–90 days), the results of the audit plan, and, if needed, recommendations for how the next A3 Reports will become standard work.

Fifty A3 Problem Solving Questions [19]–[21] populate the back side of the form and are listed as Questions S1. These questions guide the development of the project's focus and serve to remind the Author that consensus among colleagues is an essential requirement within A3 Thinking.

### III. A3 supporting documents

The Four Rules of the Toyota Production System were adopted to guide the A3 Report's Author when asking questions about the Current Condition and when designing the Target Condition [22]. Rule #1 (activities): All work shall be highly specified as to content, sequence, timing and outcome. Rule #2 (connections): Every customer-supplied connection must be direct, and there must be an unambiguous yes-or-no way to send requests and receive responses. Rule #3 (pathways): The pathway for every product and service must be simple and direct. Rule #4: Any improvement must be made in accordance with the scientific method, under the guidance of a teacher, at the lowest possible level in the organization. The 14 Principles of the Toyota Way are presented in Table 1 [23].

**Table 1:** The 14 Principles of the Toyota Way^1^.

#	Principle
**1**	Base management decisions on long-term philosophy at short-term sacrifice
**2**	Create continuous process flow in order to flush out problems
**3**	Develop pull systems that reduce overproduction
**4**	Level the workload in order to bring stability in a manner that invites standard work
**5**	Get quality right the first time by stopping to fix problems as they arise
**6**	Standardize tasks and processes in a manner that invites continuous improvement
**7**	Use visual controls in order to flush out problems in a manner that invites standard work
**8**	Use proven technology only after a clear need is thoroughly detailed
**9**	Grow leaders who thoroughly understand the work and enthusiastically teach it to others
**10**	Develop exceptional people and teams who follow the company's philosophy
**11**	Challenge and help your network of partners and suppliers to constantly improve
**12**	Go see for yourself the actual process being performed by the actual people in the actual place
**13**	Make decisions by slow, studied consensus while considering all options; implement quickly
**14**	Become a learning organization by reflecting on learnings while continually improving

1 From reference [23].

### IV. A3 teaching and coaching

A multi-session course in A3 Thinking and Problem Solving was originally developed by the Seattle Children Hospital's Continuous Performance Improvement (CPI) department with consultative guidance by Cindy Jimmerson (Lean Healthcare West, Missoula, Montana, USA). This course was subsequently revised for use at the Research Institute by Research Continuous Performance Improvement (RCPI) consultants. The Research Institute course, currently in its 9^th^ iteration, was implemented via four 1–2 hour classroom sessions spread over 2–3 months. Each student/Author was assigned a Coach, derived from a pool of RCPI consultants or from colleagues who had successfully completed the course. The Coach provided expertise in scoping, guidance during process walks and data collection, and assistance in understanding A3 Thinking and tools. Each student/Author began the A3 Report process by selecting a Sponsor (typically their supervisor) whose role was to *(i)* approve the project's commencement and implementation, *(ii)* support the Author by removing barriers, *(iii)* ensure the project's completion, and *(iv)* assure that the Followup/Audit section would be completed. Administratively, all ∼70 employees of the Institute's Research Support Services completed A3 Problem Solving Reports, to varying levels of completeness and sustainment, during FY2012.

### V. Cost estimations

In order to determine labor costs associated with *(i)* completing the A3 report and *(ii)* labor savings, a quasi-generic calculator in an Excel spreadsheet (Microsoft, Redmond, WA, USA) was used. All jobs at Seattle Children's, the parent organization of the Institute, were placed into 15 groups by the human resources department. A brief description accompanied each job group, providing additional details such as general and specific job titles and functions. After entering the number of hours contributed to the A3 report according to job group (*e.g.*, # hours for RCPI consultant, # hours for the A3 Author, # hours for the A3 Sponsor, etc.), the calculator returned a reliable estimate for the sum dollar amount of employee salary plus benefits.

### VI. Improvements via rapid Plan-Do-Check-Act (PDCA) process improvement cycles

Improvements were realized after implementation of PDCA cycles, a high level algorithm for solving problems [8]. Foundational to many quality improvement systems, PDCA cycles provide a means to *(i)* realize continuous change, *(ii)* achieve better quality in processes, and *(iii)* sustain the gains brought about by increased efficiency. Such PDCA-dependent improvements are expected to lead to stable, linear processes. After allowing for the collection of baseline data, methods for a hypothesis were developed (“Plan”) and tested (“Do”). Once metrics for the improvement had been captured, results were analyzed against the hypothesis (“Check”). If the observed outcomes failed to meet expectations, then the improvement process was revised (“Act” or “Adopt”) and retested. These cycles were repeated until the target condition was achieved.

### VII. Statistical analysis

Statistical analysis was applied to OAC A3 Report #1. The percentage of sick animal cases requiring DVM (Doctor of Veterinary Medicine) input that received DVM input within 2 hours was summarized for baseline, audit 1 and audit 2. We then compared the observed percentages using pair wise Fisher's exact tests. The threshold for statistical significance was set as *p*<0.05.

## Results

### I. The A3 Problem Solving Report is a 10-Step Scientific Method based on the P-D-C-A cycle

The 10 sections of the A3 form are aligned with Shewhart's Plan-Do-Check-Act cycle, as illustrated in Figure 1. Seven sections are devoted to Planning (“Plan”), in alignment with Principle 13 of the Toyota Way. Following *nemawashi*, the countermeasures are implemented rapidly (“Do”), in order to avoid new problems that could crop up soon after implementation. The “Check” portion of Shewhart's cycle may be the single most important part of the A3 tool, as it examines whether the desired outcomes have been attainable or not. If the desired outcomes have not been reached, then it is time to “Act” by revising the countermeasures.

### II. Overview of the A3 Problem Solving scientific method in the OAC

Nine reports were completed by OAC staff between October 1, 2011 and September 30, 2012 (Table 2). All 9 reports represented first-time efforts by the Authors in A3 Problem Solving. Authors interacted with their Sponsors prior to project initiation in order to align the problem statement with departmental and/or institutional goals. Authors “went to gemba” by visiting the location of the problem process and interacting with employees who worked the process. In cases where the problem process directly involved the Author, this was considered beneficial as the problem process was within their sphere of influence. Twice a month, Authors would congregate as a group to practice *nemawashi* by exchanging perspectives with their colleagues and interact with their Coaches. Once the left side was completed to the satisfaction of the Author, the Sponsor was asked to convey approval by affixing their dated signature to the form. The OAC group continued on to complete the right side by again interacting with each other and with their Sponsors and Coaches. Once the right side was completed, Sponsor approval was gained via dated signature and the “approved” Report was scanned and emailed to the RCPI department, which posted the Report to a SharePoint site. Authors then began their Tests, designed to measure several instances of the process over 1–2 weeks. If any of the Countermeasures failed to meet expectations, then the Author revised the “fix” (Figure 1) and the Test was repeated. Once there was an indication that a stable process had been achieved, then the Author began a 30–90 day Audit Plan. After the results of the Audit Plan were incorporated into the Follow Up section, the “final” Report was scanned, emailed to the RCPI department, which updated its SharePoint site. In this manner, the 9 OAC A3 Reports satisfied an Institutional requirement that all employees of Research Support Services would complete an A3 Problem Solving Report during FY2012.

**Table 2:** A3 Problem Solving Reports in the Office of Animal Care during fiscal year 2012.

A3 #	Title of A3 Problem Solving Report
**1**	Veterinarian input into treatment of sick animals in the Office of Animal Care
**2**	Establishing a procedure for vivarium billing with accurate activity numbers
**3**	Proper naming of mouse strains in the vivarium
**4**	Dead zebrafish report in the aquatics suite of the vivarium
**5**	Treatments of quarantine mice in the vivarium
**6**	Improve the speed of communication between vivarium staff in animal rooms and researchers
**7**	Weekend checking process of animal health and habitat in the vivarium
**8**	Overcrowding of mouse cages in the vivarium
**9**	Mouse colony organization via cage card presentation in the vivarium

Since their nine A3 Reports were focused on processes in which OAC Authors were directly involved, it followed that Authors would incorporate the Target Condition into their daily work. In most cases, Authors converted their improvements into their daily work. Presented below are two representative A3 Reports that were judged to be highly successful and sustainable.

### III. A3 Report #1: Veterinarian input into treatment of sick animals

The Issue was that the sick animal reporting process limited veterinary input into treatment options, which could impact research and was inconsistent with standard care. The Background section described that 107 sick animal cases were identified in September of 2011 by animal technicians and handled by the veterinarian technician without DVM input. A retrospective analysis determined that 52 of these cases should have received DVM input. This was the current state of the OAC because no full-time DVM was employed prior to August 2011.

**Figure 2:**
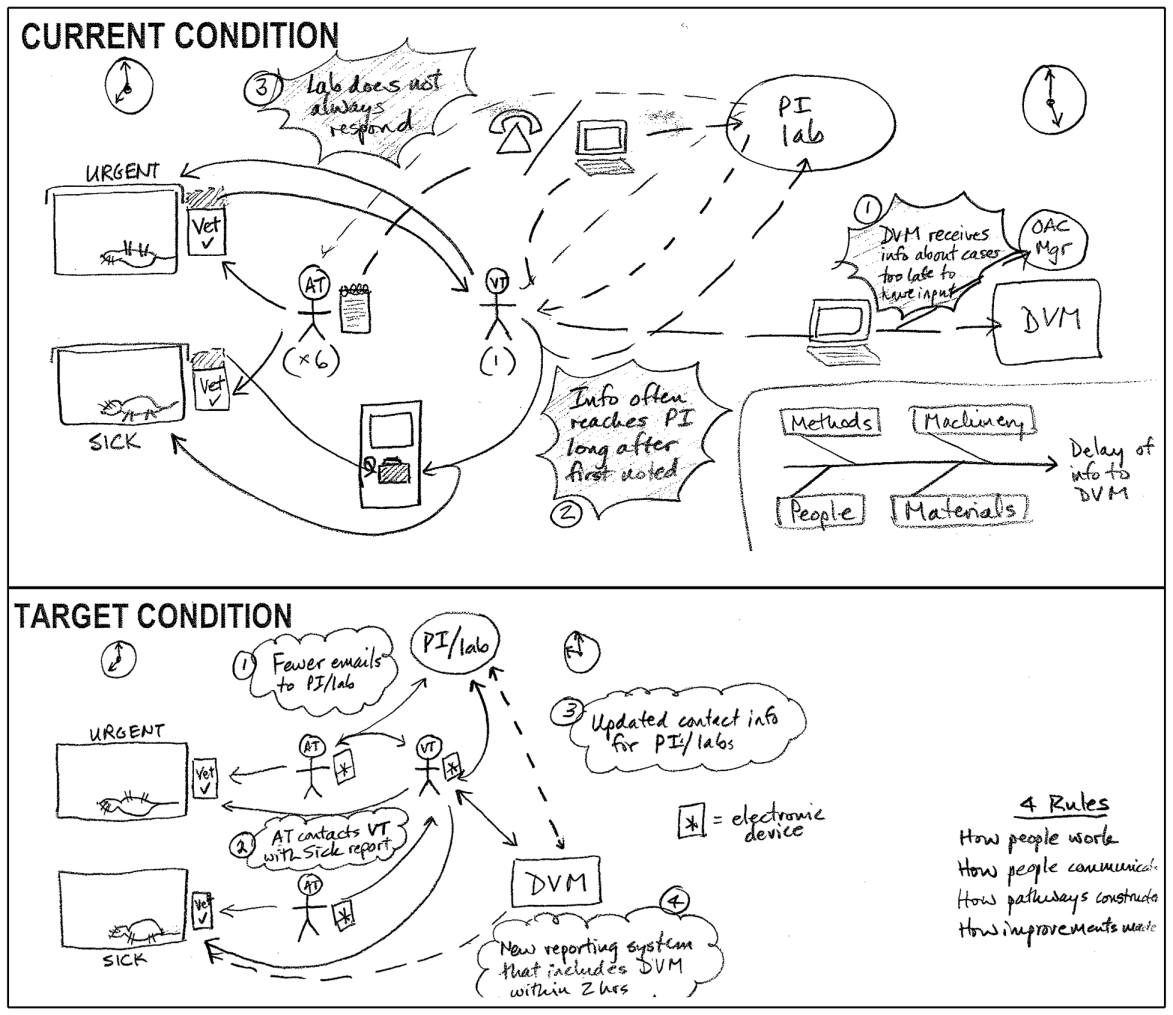
Current and Target Conditions for A3 Report “Veterinarian input into treatment of sick animals”. Animal cases are classified as either “urgent” or “sick” and are typically first detected by one of six animal technicians (AT). In each case, a single veterinary technician (VT) provides the primary interface to the researchers (PI, lab).

The Current Condition illustrated in Figure 2 tells a story of how a convoluted process notified the DVM of the work day's sick animal cases at the end of the work day, a situation that essentially prevented veterinary input into animal care. An Ishikawa diagram (lower right corner) indicated that a combination of historical Methods, communication Machinery (email, telephone), communication Materials (health/cage cards) and People (OAC staff, researchers) all contributed to delay of information to the DVM. A Five Whys Analysis (not shown) revealed that the Root Cause for the convolution was the historical work patterns that existed before the DVM was hired and that modern electronic communication tools had not been fully implemented. The following SMART Goal was therefore developed: “By end of 2011: DVM provides input into treatment of all sick animals requiring DVM consult within 2 hours of detection”.

The Target Condition (Figure 2) and Countermeasures were then developed *(i)* to reduce emails to researchers, *(ii)* for the 6 animal technicians to contact the 1 veterinary technician directly via electronic iPod iTouch (Apple Inc., Cupertino, California, USA) devices, *(iii)* to update contact info for researchers, and *(iv)* to gain DVM input within 2 hours after the first instance of sick animal detection. Costs associated with generating the A3 Report included ∼20 and ∼3 hours of DVM and OAC staff time, respectively, which was estimated by the cost calculator to be ∼$925. Expenses associated with implementing the Countermeasures included ∼$1,500 to purchase iPod iTouch devices. Expected benefits, prior to Testing, included *(i)* improved quality of animal welfare and *(ii)* improved standard veterinary care.

The quality of OAC responses to sick animal cases was evaluated prior to and after improvement, *i.e.* successful implementation of the A3 Report #1's Countermeasures (Table 3). The following two definitions of quality were evaluated: (1) *Fitness for Use* via the customer's assessment and (2) *Conformance to Specifications* via regulations established by the Institutional Animal Care and Use Committee (IACUC) and the *Guide for the Care and Use of Laboratory Animals* [24]. At Seattle Children's Research Institute, the customer is the patient/family. Data that associate customer satisfaction with DVM input into sick animal cases are non-existent, other than our assumption that the customer expects that regulations are followed during research into the cures of pediatric disease. Instead, we have assessed customer satisfaction through the eyes of the IACUC as a stakeholder, thus providing this governing body direct input into operational logistics of the OAC that influence quality. Additional stakeholders include the researcher, who designed and conducted the animal experiments, and extramural funding agencies. These stakeholders eventually have, or will, benefit from CPI-dependent improvements. After implementation, 82.4% of sick animal cases requiring DVM input received input within 2 hours – corresponding to a quality level of 1. After a PDCA cycle, the improvement rose to 90.6% – corresponding to a target quality level of 3.

**Table 3:** Levels of quality for A3 Report #1: DVM input within 2 hr of sick animal detection.

Quality Level	Quality Definition (general)	Quality Definition (OAC)	Prior to A3 improvement	After A3 improvement	Toyota Way Principle^1^
1	Customer inspects	IACUC inspects animals and OAC activity as customer's stakeholder	x		N/A^2^
2	Company^3^ inspects	Current Condition inspects during A3 Problem Solving			N/A
3	Work unit^4^ inspects	ATs and VT inspects each case for DVM relevant input		x	2,4,5,6,8
4	Self inspection	VT inspects each case for relevant DVM input			N/A
5	Mistake proofing	No relevant sick animal care occurs without DVM input			N/A

1 See Table 1.

2 N/A, not applicable.

3 company is Seattle Children's Research Institute.

4 work unit is the OAC.

During improvement work, procedures were redesigned to remove waiting and to bring flow to the sick animal process (Toyota Way Principle 2), the workflow was modified to include the DVM (Principle 4), processes were addressed such that quality was achieved the first time in gaining the timely input of the DVM (Principle 5), standardized tasks were implemented to bring stability to how the OAC responds to sick animals (Principle 6), and trusted technologies such as the iPod iTouch were brought in to enhance flow (Principle 8).

Waste removal metrics, prior to and after improvement, were also assessed. Wait time for researchers to respond to telephone calls was reduced from 2–6 hours to <2 hours (Table 4, waste #2). Multiple handling steps of sick animal information were reduced from 5 to 2 (Table 4, waste #3). Unnecessary steps, such as Animal Technicians contacting laboratory researchers directly, were eliminated through the introduction of iPod iTouch devices, a proven and reliable technology (waste #4). The number of queues in which sick animal information was communicated tallied 5 prior to improvement and 2 afterwards (Table 4, waste #5). Searching for correct researcher contact information was eliminated from the Animal Technician's role and reduced to <2 hours for the overall process (Table 4, waste #6). The defect of not involving the DVM in animal care decisions was reduced (Table 4, waste #7), as described below.

**Table 4:** Waste removal summary for A3 Report #1: DVM input within 2 hr of sick animal detection.

Waste #	Waste definition (Toyota)^1^	Waste definition (Seattle Children's)^3^	Prior to A3 improvement	After A3 improvement	Toyota Way Principle
1	Overproduction^1^	Space	N/A	N/A	N/A
2	Waiting^1^	Wait time	2–6 hours	<2 hours	2, 8
3	Unnecessary transport^1^	Transportation	# steps = 5	# steps = 2	6, 8
4	Over/incorrect processing^1^	Processing	yes	no	8
5	Excess inventory^1^	Inventory	# queues = 5	# queues = 2	2
6	Unnecessary movement^1^	Search time	2–6 hours	<2 hours	2
7	Defects^1^	Correction	yes	no	5
8	Unused employee creativity^2^	Underutilized people	yes	no	4
9	-	Complexity	N/A	N/A	N/A

1 Corresponds to the *Seven Types of Non-Value-Adding Waste* of the Toyota Production System [11], [28].

2 Corresponds to an eighth type of non-value-adding waste of Liker [28].

3 Nine types of waste identified by Seattle Children's CPI Department [27].

N/A, not applicable.

Two 30-day audits were performed to determine the number of sick animal cases requiring DVM input that actually received DVM input within 2 hours (Table 5). The baseline metric was 0%. Following several rapid PDCA cycles that optimized communication among six Animal Technicians, one Veterinary Technician, one DVM and dozens of researchers, the results of the first audit realized a significant improvement to 82.4% (*p*<0.0001). After an additional PDCA cycle, a trend towards improvement was observed (8.2% to 90.6%, *p* = 0.11).

**Table 5:** Baseline and follow up metrics for A3 Report #1: DVM input into treatment of sick animals.

Metric type with dates	Sick animal cases requiring DVM input % (#)	Sick animal cases requiring DVM input that received DVM input within 2 hour % (#)
Baseline (prior to Sept 6, 2011)	49 (52/107)	0
Audit 1 (Feb 15–March 15, 2012)	59 (119/203)	82.4 (98/119)
iPod iTouch devices tested for reception in animal rooms	N/A^2^	N/A
iPod iTouch devices purchased for ATs and VT^1^	N/A	N/A
ATs and VT use iPod Touch devices to send sick animal case info, including weekends	N/A	N/A
Audit 2 (May 15–June 15, 2012)	57 (85/148)	90.6 (77/85)

1 AT, animal technician; VT, veterinary technician.

2 N/A, not applicable.

### IV. A3 Report #7: Weekend checking process of animal health and habitat in the vivarium

The Issue focused on the confusing process to keep track of what has been completed and not completed during weekend/holiday checks. Such checks involved animals, feeds, treatments and reports – the types of activities that could be fit into an 8–10 hour shift by one OAC staff individual. The significance of this problem was that, if left unsolved, the obligation of not meeting IACUC expectations could be compromised.

As Background, the vivarium at SCRI consists of 22 rodent, 2 large animal, 4 fish and 3 satellite animal rooms spread over multiple floors in the same building. Responsibilities involve an overall health check of animals, mechanical functionality, and the feeding and treatment of animals. The Current Condition described a linear process where the OAC staff individual could be called away for emergencies or to procure treatment medications, thus triggering a lapse in where the checking left off and where it needed to be picked back up. As the Institute grows by adding investigators who use animal models in their research, a true need for optimization of the weekend/holiday check is essential.

A Five Whys analysis concluded that the Root Cause of the dysfunction was that the OAC had variation in the process for weekend/holiday checks. Accordingly, the Goal of this A3 Report was to “create/maintain a process to find where staff had left off in the routine facility check and know what had been completed/not completed”.

The Target Condition and Countermeasures specified *(i)* the creation of a “Weekend Check Log” that contained all responsibilities in reusable, laminated check forms that clearly delineated a standard process of checking, *(ii)* training of OAC staff in use of the log, and *(iii)* maintaining an updated log for all weekend/holiday checks.

Engagement with OAC staff by the Author was via *nemawashi*. The Countermeasures were carefully planned through 8 Implementation Steps and then rapidly implemented (“do”), in alignment with Shewhart's Plan-Do-Check-Act cycle. The Cost of improvement was minimal (7 hrs of staff time) while the Cost Benefits were organizational, time management and 1–2 hours of time saved each holiday or weekend day.

Testing of the improvement took place over a 2-day weekend and identified minor problems and inconsistencies. A rapid PDCA cycle was performed, followed by re-testing over the next weekend. After another round of rapid PDCA, the improvement was subjected to a 73-day audit period that spanned 10 weekends and 1 holiday. After a final round of rapid PDCA, the improvement was incorporated into standard work for all OAC staff assigned to such responsibilities.

Because, in part, of the quality and pace at which this Author pursued this A3 Problem Solving Report, he was promoted to be an OAC CPI team leader in a manner consistent with Principle 9 of the Toyota Way: “Grow leaders who thoroughly understand the work, live the philosophy, and teach it to others.”

## Discussion

The usefulness of the A3 Problem Solving Tool has been validated as the OAC undergoes a productive transformation, in alignment with Principle 14 of the Toyota Way: “Becoming a lean organization through relentless reflection (*hansei*) and continuous improvement (*kaizen*).” Being able to complete 9 A3 Problem Solving Reports in a manner that utilizes rapid PDCA cycles and downstream incorporation into daily standard work is a solid foundation as the OAC breaks down silos and implements cross-training. The OAC, as a department within Research Support Services, has begun its CPI journey in a manner consistent with the following 4P Model developed in *The Toyota Way* [25]. Philosophy: Dr. Ida Washington, OAC Director, and Dr. James Hendricks, Research Institute President, view the OAC as a means to adding value to patients of Seattle Children's Hospital – namely supporting research into cures of pediatric diseases and conditions. People and Partners: Dr. Washington has team-empowered the OAC staff in the department's CPI journey. These nine A3 Problem Solving Reports certainly support the Research Institute as a learning organization. Process: Elimination of waste in the OAC has been accomplished by applying CPI principles such as A3 Reports and standard work. Problem solving: The CPI toolbox to solve problems within the OAC is growing and currently consists of A3 Reports, 5S organizing systems, a Kaizen board, a Daily Management System, and Heijunka and pitch boards.

Implementation of the Countermeasures in an A3 Problem Solving Report is often expected to lead to incorporation into standard work. For OAC A3 Report #7 (“Weekend checking process of animal health and habitat in the vivarium”), the weekend check log is now a stable of standard work on weekends and holidays. The log is described in the standard work document for OAC huddles, and is discussed on the day prior to and after its use. Typical data that populate the log include special feeding or handling instructions for any given animal, technical procedures such as injections, or logistical considerations in the aquatic center. The collective data indicate that A3 Thinking has taken hold for A3 Report #7 in a manner aligned with Principle 14. Consistent with this A3 Thinking is a new PDCA proposal to convert the written log into an electronic log in order to reduce errors attributable to handwriting legibility issues.

The format of A3 Problem Solving Report form that is currently in use at the Research Institute lacks sufficient white space for documentation of observed outcomes (Figure 1, step 7) and for the results of the testing process (Figure 1, step 9). This brevity contrasts with the typical results section of a scientific manuscript, which is the most important part of peer-reviewed, published studies. While this brevity is probably founded on the notion that A3 Thinking is paramount to the A3 Problem Solving Report, there is no formal restriction that prevents appending additional outcomes and results to the Report – other than the overall requirement to present the entire Report on one side of an 11×17 inch piece of paper. The Author is faced with aligning these results with the basis for comparison that is stated in the Goal section (Figure 1, step 4). It then follows that A3 Thinking provides a platform for establishing a causal linkage between the action items of the Countermeasures and the outcomes of the Implementation Plan. In this perspective, the A3 Problem Solving Report does align quite well with traditional hypothesis-driven scientific experimentation.

A conventional position in laboratory animal care is that compliance equals quality. But that attitude is misplaced, especially in the new Guide's allowance for performance standards that permit flexibility in designing and evaluating evidence-based approaches for desired outcomes [24]. Being able to implement CPI-dependent improvements that specifically address Toyota's definition of quality can only serve to benefit the OAC's stakeholders: the IACUC, the researcher and the extramural funding agency. The ultimate beneficiary, of course, is Seattle Children's customer – the patient/family who awaits new cures for pediatric disease.

Veterinary medical care is an essential part of any animal care and use program. Such a program includes, at a minimum, effective plans for preventive medicine, monitoring and treatment of disease, surgery and post-op care, and anesthesia, analgesia and euthanasia [24]. Given that parts of each plan can be carried out by OAC staff, the communication within the OAC during the assessment and treatment of sick animals becomes paramount. Likewise, the daily observations of animals for signs of illness, injury or abnormal behavior need to be conducted by trained personnel. Such observations include holidays and weekends, a process in which a single OAC staff member is expected to visit every cage in the facility – a somewhat daunting task that was standardized by OAC A3 Report #7.

Being able to reduce the time it takes for veterinary input in sick animal cases has brought the OAC closer to realizing one-piece flow for that process. An important assessment of the sick animal Current Condition (OAC A3 Report #1) was to identify what was value added, using the Three Toyota Categories: *(i)* value added, *(ii)* non-value added and *(iii)* non-value added but required [26]. In the context of waste, Seattle Children's lists the following three categories: *(i) muda*, non-value added, *(ii) mura*, unevenness or variation in work processes, and *(iii) muri*, overburdening people or equipment [27]. Seattle Children's teaches that there are 9 types of waste contained within the *muda* category (see Table 4). Step by step, the OAC is pursuing its True North by acting as a tortoise and not the hare. Removal of waste can only lead to increased efficiencies while allowing OAC staff to increase supportive interactions with Institute researchers. Lean improvements do not happen overnight. Instead, they are a transformative process – and for a complex healthcare organization like Seattle Children's, this CPI journey is expected to demonstrate constant planning, doing, checking and acting.

## Supporting Information

Questions S1 **Fifty A3 Problem Solving Questions [19]**–**[21] populate the back side of the A3 form.** These questions guide the development of the project's focus and serve to remind the Author that consensus among colleagues is an essential requirement within A3 Thinking.
(PDF)
