# Supplementary material for: The A3 Problem Solving Report: A 10-Step Scientific Method to Execute Performance Improvements in an Academic Research Vivarium
Source: PLoS One. 2013 Oct 29;8(10):e76833. doi: 10.1371/journal.pone.0076833 (PMC3812205; doi:10.1371/journal.pone.0076833)
Supplement: Questions S1 — Fifty A3 Problem Solving Questions [19]–[21] populate the back side of the A3 form. These questions guide the development of the project's focus and serve to remind the Author that consensus among colleagues is an essential requirement within A3 Thinking. (PDF) [file pone.0076833.s001.pdf]

**Questions S1** A3 Problem Solving Questions[1]

The A3 Problem Solving Report: A 10-Step Scientific Method to Execute Performance Improvements in an Academic Research Vivarium. James A. Bassuk, PhD and Ida M. Washington, DVM, PhD, DACLAM

**LEFT SIDE**

**Issue**

1. Does the issue give a good description of what the report is about?
2. Is the issue or theme stated as it relates to the customer/patient (not the workers)?
3. Does the statement appear causal or blaming? Does it advocate for a solution?
4. Does the scope appear appropriate for an A3, or is it too broad? (If too broad, can it be broken into smaller issues?)
5. Is the issue within the Author's (or Sponsor's) area of influence?

**Background**

6. Do you have enough content to understand how the issue aligns with the organization and goals?
7. Do you understand the history of the problem? Who is affected by the problem?
8. Do you understand why the problem is important?
9. How is the problem quantified? How often does it occur? Can you assign a cost?
10. Is there any other reason for working on this topic? (e.g. learning purposes?)

**Current Condition**

11. Can you easily and quickly grasp how the current process works?
12. Are the facts of the situation clear and based on observations/data versus opinions?
13. Do the issue statement and current condition directly relate to one another?
14. Has the Author gained consensus on the current condition from those performing the work?
15. Are the problem areas identified with bursts?

**Goal Statement**

16. Does the goal statement describe the outcome you are working to achieve?
17. How will performance be measured?
18. What standard or basis for comparison will be used?

**Root Cause Analysis**

19. Has the Author identified the most important problems to address for this issue?
20. Do the problems addressed correspond to a storm cloud in the current condition?
21. Has the analysis gone to enough depth? Are the roots of the problems clearly and sufficiently identified?
22. Is there evidence of proper five-whys thinking about the true cause?
23. Have the problems been identified as: a) failure to specify an activity, b) ambiguous, complex or non-existent connections, c) complex pathways or d) lack of response to a problem?
24. Has cause and effect been demonstrated or linked in some manner?
25. Are all the relevant factors considered (human, machine, material, method, environment, measurement, and so on)?

**RIGHT SIDE**

**Target Condition**

26. Can you easily and quickly grasp how the new process will work?
27. Does the new target condition address the root cause of the problem?
28. Have features been identified with fluffy cloud bursts? Are they SMART?
29. Does the proposed target condition move the organization closer to ideal? (exactly what the patient needs – defect free, immediate, on demand, one-at-a-time, without waste, safe for all)

**Countermeasures**

30. Are there clear countermeasure steps identified?
31. Do the countermeasures link to the root cause of the problem?
32. Do they define what needs to happen to move us from the current condition to the target condition?
33. Will they prevent recurrence of the problem?

**Implementation Plan**

34. Does the plan cover the set of countermeasures identified in the target condition?
35. Has each activity in the plan been specified as to who, what, when and outcome?
36. Is the plan realistic and achievable? Is the scope of proposed work realistic for the time assigned?
37. Do the points in the plan eliminate the root causes identified in the problem analysis?
38. Are the people involved with the implementation plan aware of their commitments?
39. What other parts of the organization need to be informed of this result?

**Cost/Cost Benefit**

40. Have you determined the cost of the implementation plan?
41. Can you predict, then measure, cost/benefits to be realized?
42. Can you assign dollar value to the waste identified?
43. Is the net gain clearly stated? What is the return on investment (ROI)?

**Test**

44. Is the test designed as an experiment?
45. Is the pilot well thought out and defined?
46. Does the test predict measurable results?

**Follow-up**

47. Do you know when the audits will occur?
48. How do the actual results compare to anticipated results?
49. Is there a plan to sustain and hold the gains?
50. Have the problems been addressed? Have other problems surfaced that you need to continue with another A3?

[1]Abstracted from references [19–21]
